# Understanding how whānau-centred initiatives can improve Māori health in Aotearoa New Zealand

**DOI:** 10.1093/heapro/daad070

**Published:** 2023-07-13

**Authors:** Angelique Reweti

**Affiliations:** Massey University, College of Health, Private Bag 11 222, Palmerston North 4442, New Zealand

**Keywords:** community health promotion, capacity building, health promotion, Indigenous health, Māori health and wellbeing

## Abstract

This article highlights the significance of prioritizing Indigenous voices and knowledge systems, using whānau-centred initiatives (a concept that encompasses the broader family and community) as a foundation for health promotion within an Indigenous context. Tū Kahikatea, a conceptual framework, is used to demonstrate the relationship between the values underpinning different whānau-centred initiatives and their corresponding outcomes. The framework highlights the capacity of whānau-centred initiatives to support whānau in attaining mana motuhake, which represents collective self-determination and the ability to exercise control over their own future. By doing so, these initiatives contribute to the improvement of whānau health outcomes. With recent changes to Aotearoa New Zealand’s health system, the findings underscore the benefits and potential of whānau-centred initiatives in enhancing whānau health outcomes, and advocate for continued strengths-based practices in Aotearoa New Zealand’s health system. By bridging the gap between academia and grassroots community action, the article demonstrates the potential of whānau-centred initiatives and contributes to a global call for integrating Indigenous viewpoints and practices into Westernized healthcare, in order to improve Indigenous health outcomes.

**Contribution to Health Promotion**

- Indigenous voices and knowledge are key to addressing health inequities experienced by Indigenous peoples.
- Māori values and practices are the foundation of whānau-centred initiatives in Aotearoa New Zealand.
- The Tū Kahikatea framework shows how whānau-centred initiatives can enhance health outcomes and achieve collective self-determination.
- Aotearoa New Zealand's changing health system presents opportunities for whānau-centered initiatives to build capacity and improve health outcomes.
- Service design and delivery should focus on the needs of whānau and those who use them to improve Indigenous health outcomes.

## INTRODUCTION

A global call has been issued to prioritize Indigenous voices and Indigenous knowledge systems in health promotion (IUHPE, 2019; Ratima *et al.*, 2019; Walters *et al.*, 2020; Watego *et al.*, 2021). Indigenous health promotion serves as a bridge between Indigenous development and general health promotion, giving Indigenous peoples a vehicle to realize their hopes for a healthy, proud and self-assured future (Durie, 2004; Ratima *et al.*, 2019). Despite differences in historical context and cultural orientation, the concepts of health and wellbeing among Indigenous peoples are embedded in their worldview, culture and language. These notions are influenced by their pursuit of self-determination, diverse identities, aspirations and the complex realities shaped by colonization (Ratima *et al.*, 2019; Redvers *et al.*, 2020; Walters *et al.*, 2020; Reweti *et al.*, 2022). Indigenous health promotion frameworks uphold a holistic and relational approach to wellbeing including spiritual and social aspects alongside the more biomedical focus of physical and mental wellbeing. The centrality of relationships with the environment to health and wellbeing is emphasized, as well as the significance of having meaningful opportunities to engage in activities that secure cultural identity (Panelli and Tipa, 2007; Kingsley *et al.*, 2009; Tu’itahi and Lima, 2015; Redvers *et al.*, 2020; Severinsen and Reweti, 2021). Consistent with values, aspirations and self-determination of the people, an Indigenous health promotion approach focuses on building community strength and resilience (Durie, 2004; IUHPE, 2019; Walters *et al.*, 2020). This type of approach facilitates healthy lifestyles while also strengthening cultural identity and the spiritual connection between wellbeing and the environment (Durie, 2004; Kingsley *et al.*, 2009; Tu’itahi and Lima, 2015; Redvers *et al.*, 2020; Severinsen and Reweti, 2021). An Indigenous approach to health promotion also recognizes the importance of focussing on the collective community as opposed to focussing on individuals (Tu’itahi and Lima, 2015; Redvers *et al.*, 2020; Walters *et al.*, 2020).

From a te ao Māori (the Māori world) perspective, health and wellbeing are based on connections and the concept of whanaungatanga (process of forming and maintaining relationships) with an understanding that health extends beyond that of individuals, disease and illness (Wilson *et al.*, 2021b). The term ‘hauora’, commonly used to describe Māori perspectives on health, is a holistic concept that encompasses all dimensions of a person, including their spirituality and cultural identity, alongside physical and mental wellbeing. If we break down the kupu (word), one of the meanings of hau is the vital essence embodied in all persons and living things according to a Māori worldview (Henare, 2001; Moorfield, 2021). An expression of hau in people is breath, while ora is about health, life and vitality (Moorfield, n.d. a). Therefore, ‘hauora’ can be understood as representing the complete set of vital elements that contribute to a dynamic and thriving spirit of life (Reweti *et al.*, 2022).

Concepts such as mauri and mana help to explain a Māori view of hauora (Reweti *et al.*, 2022). Mauri is the physical manifestation of hau binding the wairua (spiritual element) and tinana (physical element), making it the very essence of life (Henare, 2001). Mauri permeates all things, both animate and inanimate, including people, the environment, buildings, space and time, all of which are interconnected and have a direct impact on one another with the mauri of one thing influencing the mauri of another (Henare, 2001; Marsden and Royal, 2003). An understanding of mauri encourages people to pay attention to the vitality of their energy, the factors that motivate them to act and interact with the world around them and emphasizes the need for balance and harmony between all living things (Penehira *et al.*, 2011). Mana is about spiritual vitality and is often described as power, authority or prestige (Barlow, 1991). There are many manifestations of mana and our experiences of it, such as mana tūpuna, a person’s inherited authority linked to whakapapa (genealogy), mana whenua, the authority derived from historic and territorial rights over land, and manaakitanga, the way in which mana is harnessed through generosity and empathy to look after one another (Mutu, 2020). Another manifestation of mana is mana motuhake, which refers to Māori exerting their authority over their lives and living on their own terms and in line with Māori philosophy, beliefs and customs, which is viewed as crucial in realizing health and wellbeing for a people (Mutu, 2020; Oetzel *et al.*, 2020).

Despite broad awareness of Indigenous health status and increased study into risk factors, health inequities between Indigenous and non-Indigenous peoples continue to exist (Wilson *et al.*, 2021c). In Aotearoa New Zealand, the recent Wai 2575 report (Waitangi Tribunal, 2019) details a litany of health inequities between Māori and non-Māori that stem from colonial experience alongside a health system that systematically fails to address the needs of Māori. Individual risk factors aligned with a conventional approach to health promotion are emphasized overlooking a more holistic perspective associated with a Māori view of health and wellbeing (Wilson *et al.*, 2019). Furthermore, there has traditionally been a top-down approach to health efforts in Aotearoa New Zealand, limiting Māori participation in the design and implementation of services and strategies that directly affect their lives. Many health promotion strategies are targeted at addressing the risk factors of an individual’s or community’s health status, such as obesity, smoking and diabetes, and take a deficit approach to health (Warbrick *et al.*, 2016; Wilson *et al.*, 2019).

A deficit approach refers to a way of thinking that often frames and represents Indigenous peoples through the perspective of negativity, inadequacy and failure (Fogarty *et al.*, 2018; Watego *et al.*, 2021). This type of approach, which typically privileges Western forms of knowledge and existence over Indigenous forms (Bryant *et al.*, 2021), exacerbates the marginalization of Māori voices, attitudes and worldviews. Such an approach frequently overlooks the larger socio-economic structures within which inequities are embedded, with disadvantage becoming so established in reductionist narratives of failure that Māori are frequently viewed as the source of the problem. Critiquing a deficit approach is not about ignoring the well-documented realities of disadvantage in health experienced by Māori (Palmer *et al.*, 2019; Reid *et al.*, 2019) however, only looking at a situation from a deficit perspective restricts the ability to see other possibilities that could lead to sustained growth and beneficial change in health and wellbeing.

A strengths-based approach emphasizes the unique characteristics of individuals and communities, rather than stereotypes and deficiencies. It encourages the use of capabilities and resources to heal and become empowered, acknowledging that everyone possesses a variety of qualities and strengths that can be leveraged with support for a more fulfilling future. This approach does not deny the existence of difficulties or challenges but rather accepts that individuals can become resilient and resourceful through experiencing hardship and learning to overcome adversity (Pulla, 2012; Fogarty *et al.*, 2018). It also recognizes that personal characteristics, as well as social and cultural factors, influence wellbeing and that people need to be involved in determining goals to build on their own strengths (Pulla, 2012; Fogarty *et al.*, 2018).

Whānau ora exemplifies a strength-based approach, reframing Māori experiences and outcomes in a positive light. Practiced by Māori for generations, this philosophy empowers whānau as a whole, recognizing their inherent capacity to learn, grow and transform. It focusses on strengths and aspirations rather than deficits, upholding the concept of mana motuhake, where whānau have the ability to define their own problems and devise solutions. While the circumstances in which contemporary whānau live are diverse, encompassing a variety of social, economic and cultural contexts, the collective responsibility that whānau share remains a unifying factor (Durie *et al.*, 2010; Lawson-Te Aho, 2010). Rather than a community passively waiting for top-down public health interventions, many whānau are actively pursuing health and wellbeing through several different whānau-centred initiatives. These include activities such as Iron Māori which supports inclusive multisport events catering to a range of fitness levels (Pohatu, 2015; Jones *et al.*, 2020); the Muriwai Sports Tournament combining sporting activities with a chance for whānau to reconnect to their ancestral heritage; whānau wānanga (discussion forum) held by the PS Haitana Whānau Trust centred around finding ways to more effectively respond to mental health issues within their whānau (Savage *et al.*, 2019); Wero Warrior instigated by the S J Pikia Family Trust that supports whānau to overcome barriers that have affected their ability to lead and manage healthier lifestyles (Savage *et al.*, 2020); and Awa Ora created by whānau at Whakatū Marae (ceremonial meeting house) which focuses on whānau cleaning up the local awa (river) to lift and restore the wairua and mauri of the whānau (Savage *et al.*, 2018). These whānau initiatives highlight the strength and resourcefulness of whānau and the ways that communities of people can work together to improve collective wellbeing.

Aotearoa New Zealand is currently undergoing significant health reforms. A centralized health agency (Te Whatu Ora, Health NZ) has been established to replace 20 District Health Boards, as well as the development of an independent Māori Health Authority (Te Aka Whai Ora) to lead and monitor transformational change for the health and wellbeing needs of whānau Māori (Came and O’Sullivan, 2021; Eggleton *et al.*, 2021). The partnership between the Māori Health Authority and Health NZ aims to invest in services grounded in te ao Māori and ensure the wider health system recognizes and is more responsive to Māori needs, alongside that of the wider population (Te Aka Whai Ora, 2023). This paper presents three case studies showcasing diverse whānau-centred (family/community centred) initiatives that highlight the essential role of whānau in improving health and wellbeing of Māori. According to the findings, initiatives developed within whānau communities and based on Māori worldviews and values are more likely to positively influence Māori health outcomes than top-down, single-issue, time-limited health promotion programmes.

## METHODS

Mātauranga (Māori epistemologies/ways of knowing) informed the research practices used in this study, which prioritizes Māori ways of knowing and doing, as well as adopting techniques that take full cognisance of tikanga (Māori principles that inform practice) (Mead, 2012; Durie, 2021). Mātauranga is rooted in the spiritual health, culture and language of the people and cannot be compartmentalized or separated from the people who hold it (Royal, 2009, 2011; Doherty, 2012). Rather than dissecting knowledge into smaller pieces, mātauranga emerges from the interweaving of numerous sources, where links to greater dimensions and layers of knowledge are established (Durie, 2021). In this way, knowledge creation can be likened to the process of raranga (weaving) with each source of information symbolizing a single rau (leaf) that is woven together to create something new.

The practice of raranga entails meticulous preparation and embodies essential qualities like commitment, patience and creativity, all of which align with the process of knowledge creation from a te ao Māori perspective (Reweti *et al.*, 2022).

To demonstrate the potential benefits of a whānau-centred approach to aiding Māori health and wellbeing, this article synthesizes results from three case studies about whānau-centred initiatives. As a platform for narratives to be heard, evaluated and studied, case studies permit in-depth investigation of an event or phenomenon (Crowe *et al.*, 2011; Yin, 2014). Instead of using a single example to analyse and comprehend phenomena, a multiple case study approach makes use of a collection of cases to evaluate what those cases might reveal about the broader context of that issue (Crowe *et al.*, 2011; Yin, 2014). Collectively, the case studies provide insight into a diversity of whānau-centred initiatives. Each of the original case studies focus on health promotion within an Indigenous context, where Māori values and practices are foundational. The research focussed on exploring the social, cultural and health benefits of whānau-centred initiatives.

Case Study 1 focuses on an initiative established by a whakapapa (genealogical) whānau. Case Study 2 is an example of a kaupapa whānau initiative originating from within the community, while Case Study 3 was instigated through the support of a local District Health Board and a community sporting agency. In-depth articles about each of the case studies, including two short films based on Case Studies 1 and 2, can be found elsewhere (Reweti, 2019, 2022a,b; Severinsen and Reweti, 2020, 2021). Ethics approval for the case studies were granted through a University Ethics Committee and guided by a kaupapa whānau ethics framework developed by the researcher alongside whānau involved in Case Study 1.

Case studies were analysed thematically from a te ao Māori perspective, using topic categories related to social, cultural and health benefits. Recurring patterns, themes and concepts across the case studies were identified and examined, with a focus on Māori values and worldviews. The goal of this approach was to centre and highlight whānau perspectives and provide insights into their experiences, while also allowing space to acknowledge the diversity and dynamic nature of these different experiences. Collaboration with whānau was necessary to ensure that the analysis was culturally appropriate and meaningful. This resulted in the expansion of Tū Kahikatea (which was originally developed during the analysis of Case Study 3) as a framework for depicting the relationship between core principles and related outcomes of whānau-centred initiatives, as well as how this translates into whānau achieving mana motuhake.

**Fig. 1:**
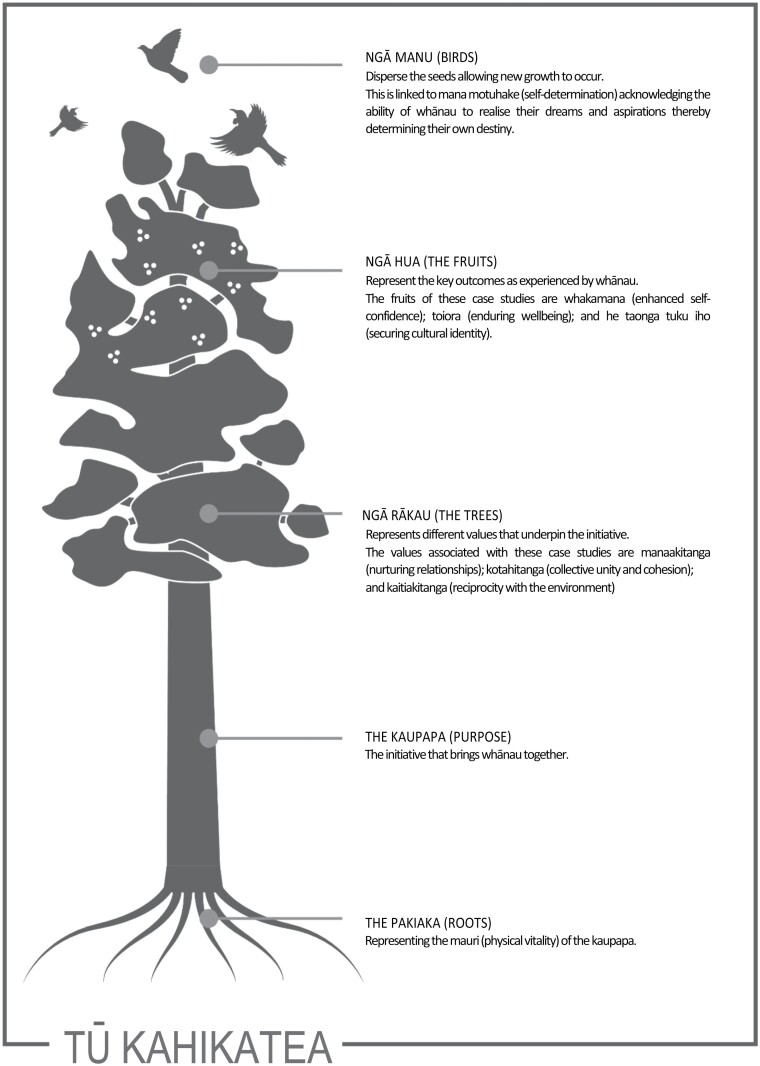
Tū Kahikatea framework.

Tū Kahikatea (Figure 1) is a framework that represents the importance of whānau and whanaungatanga in supporting health and wellbeing. Whanaungatanga is generated through shared experiences, a sense of belonging, and reciprocal rights and obligations. Likewise, the roots of Kahikatea trees are intertwined by standing close together, allowing them to fully withstand any pressure that may be placed on them individually and collectively. The framework is composed of five core components: pakiaka (roots), representing physical vitality; kaupapa (purpose), requiring a unifying purpose; ngā rākau (the trees), representing different core values; ngā hua (the fruits), representing the outcomes experienced by whānau; and ngā manu (the birds), which disperse the seeds allowing new growth and self-determination (Reweti, 2019).

### Case Study 1: Reweti Whānau Hui

Instigated by whānau in 2012, the Reweti Whānau Hui (RWH) provides an opportunity for whānau to strengthen connections with their ancestral marae and whenua (land) and to engage in te ao Māori while fostering and developing bonds of unity amongst extended whānau members. Together the whānau have been learning about their whakapapa and connections to the whenua and each other through learning their pepeha (form of introduction establishing identity), through waiata (song) and through haka (ceremonial dance or challenge), which in turn also increases their confidence using te reo (Māori language) (Reweti, 2022a, b). Another key kaupapa of the RWH is the chance for members of the whānau to discuss what they have been focussed on since their previous hui and to recognize and celebrate their successes. Data for this project were collated from 16 participants ranging in age from 15 to 80 years through kanohi ki te kanohi (face-to-face) interviews and video footage obtained from filming one of the RWH.

#### Impact

With most of the Reweti whānau living in an urban context away from their ancestral roots, the whānau hui has provided a safe environment for whānau to explore and further secure their cultural identity as Māori. Te reo Māori me ōna tikanga (Māori language and its cultural practices) has provided an environment for intergenerational learning where values and skillsets important to the whānau are passed down (Reweti, 2022a, b). Through whānau role modelling, values such as aroha (love/compassion), kotahitanga (collective unity), manaakitanga (generosity and caring for others) and tuakana teina (mentorship) are all interwoven into the day-to-day experiences of being on the marae. The collective mana of the whānau has been enhanced through fostering physical, spiritual and emotional connections with each other, their cultural identity, their whenua and their marae (Reweti, 2022a, b). The RWH is an example of whānau exercising mana motuhake by developing their own aspirations and devising strategies to achieve them, providing an environment where whānau identity can be actively expressed and cultivated, and where for this whānau, being Māori is now celebrated.

### Case Study 2: Waka Ama (outrigger canoe club)

Case Study 2 involves research with a local waka ama rōpū (outrigger canoe club) from Heretaunga, Aotearoa New Zealand. Waka ama (outrigger canoe) is an increasingly popular sport in Aotearoa New Zealand utilizing Māori values and beliefs to improve the health of individual paddlers, their whānau and communities (Severinsen and Reweti, 2021; Reweti and Severinsen, 2022). A key component of the success of Waka Ama is the active involvement of members of the local community that generously contribute their time and energy to support the group’s activities and participation in club and national competitions. In addition to the physical act of paddling, Waka Ama provides opportunities for involvement in governance, kaitiakitanga (guardianship) activities and leadership and educational development (Reweti and Severinsen, 2022). Sixteen participants were directly involved in the research through a series of loosely structured conversational kanohi ki te kanohi (face-to-face) interviews and video footage of paddlers obtained from following two morning training sessions (Severinsen and Reweti, 2021).

#### Impact

As well as offering physical health benefits to paddlers, waka ama fosters cultural identity, social connectedness, intergenerational participation and community cohesion (Severinsen and Reweti, 2021; Reweti and Severinsen, 2022). Involvement encouraged the use of te reo through karakia (incantations) and waiata and learning about tikanga associated with waka ama. Many participants have taken advantage of the opportunity to increase their knowledge of tikanga and further their education through courses offered by local institutions. By fostering a reciprocal relationship between paddlers and the environment, waka ama encourages members to recognize their responsibilities as kaitiaki in caring for the environment (Severinsen and Reweti, 2021; Reweti and Severinsen, 2022). This has led to increased involvement in conservation efforts. The culture of waka ama also promotes healthy lifestyle choices by encouraging smoke-free, alcohol-free and fizzy (sugar sweetened beverage). Paddlers have reported increased self-confidence due to improved physical ability, with links to improved mental wellbeing.

### Case Study 3: Sport Manawatū WhānauTri

Instigated in 2013 in partnership with the local MidCentral District Health Board, WhānauTri is a community-based physical exercise and health initiative, created for whānau to become more active through a 10-week programme that culminates with a whānau triathlon (Sport Manawatū, 2019). The programme’s purpose is to teach whānau how to train for and complete a triathlon, as well as how to create long-term lifestyle changes by improving their knowledge of physical fitness, nutrition and goal-setting abilities. On the day of the event, a vibrant festival atmosphere is created to celebrate both health and culture. The festivities include health-related stalls, entertainment, spot prizes and a wide range of additional activities and competitions for whānau to partake in and enjoy (Reweti, 2019). Encouraging participation across all age groups, the WhānauTri has become an annual event for many whānau in the Manawatū region which includes intergenerational teams of kaumātua (grandparents), mātua (parents), whaea kēkē and mātua kēkē (aunts and uncles), tamariki (children) and mokopuna (grandchildren) (Reweti, 2019). Data for this project came from engagement with six whānau groups incorporating 30 whānau members.

#### Impact

The WhānauTri programme fostered connections among whānau, strengthening interpersonal relationships and creating a sense of social responsibility. It facilitated opportunities for whānau to expand their networks of support and engage in outdoor activities, leading to positive lifestyle changes and increased wellbeing. Many participants experienced their first positive sporting and health experience through the programme, boosting their self-esteem and self-worth. By the end of the programme, whānau felt confident in their abilities to engage in local community resources and other hauora activities (Reweti, 2019). The WhānauTri served as a catalyst for pursuing other healthy lifestyle ventures and realizing their dreams, with participants citing it as a motivating factor for expanding their vision of what was possible.

## FINDINGS

These case studies illustrate several commonalities that are implicit in a whānau-centred approach. Each of the case studies provided opportunities for intergenerational engagement strengthening interpersonal relationships and social cohesion between whānau members and/or within the community. Whānau were able to develop a sense of connection to te taiao (the natural environment), as well as learn about and actively participate in a reciprocal relationship with the environment. Whānau spoke about increased levels of self-confidence which positively impacted on their physical and mental wellbeing. Two of the initiatives provided a gateway into te ao Māori, fostering strategies for securing cultural identity. Achieving sustainable lifestyle gains was established in all case studies. Initiatives gave whānau the opportunity to assume leadership roles and/or be a part of creating and working towards achieving their own personal aspirations. These findings are aligned with Māori concepts of manaakitanga, kotahitanga, kaitiakitanga, whakamana (empowerment), he taonga tuku iho (ancestral treasures passed down through the generations), toiora (enduring wellbeing) and mana motuhake. Using Tū Kahikatea framework as a guide, findings are discussed in conjunction with verbatim comments collected from whānau as a means of reinforcing central ideas.

### Pakiaka (roots): represent the mauri (life force) of the kaupapa

Mauri is the life spark or essence that binds and animates everything in the physical world (Henare, 2001). It underpins the Tū Kahikatea framework to indicate that all living things, including our endeavours, have a mauri that influences and is influenced by the surrounding environment. In this context, mauri embodies the energies required to fulfil the interests, objectives and aspirations of those involved in these whānau-centred initiatives.

### Kaupapa (purpose): represents the purpose that brings whānau together

Kaupapa refers to the collective vision, aspiration and purpose of the whānau-centred initiatives. While each of the three case studies in this research are diverse, they all focus on strengthening whānau capability and advancing whānau towards improved health and wellbeing.

### Ngā rākau (the trees): represents overarching values underpinning initiatives

Core values of manaakitanga, kotahitanga and kaitiakitanga are evident in all of the case studies. Manaakitanga is about nurturing relationships and is concerned with the protection of a person’s mana through acts of kindness, support and encouragement (Mead, 2003; Mutu, 2020). A fundamental principle of manaakitanga is cooperating with others in a spirit of reciprocity, holding everyone to a high standard of conduct towards one another. Examples of manaakitanga in practice are expressed in the following statements from whānau:

It’s a very pleasant experience to come here and unplugging, and getting back to core values and traditional tikanga, for example, we had young boys 10 to 14 preparing food for their elders and as simple as that is, it instils a core value that we really want our children, the next generation, to firstly just be good people, and what greater way to teach that then through service, so that’s one of the fundamental tenants of tikanga Māori, is manaakitanga, or taking care of people, so as simple an action of gutting and scaling a fish, and cooking, then serving it, then doing the dishes, those are the types of things that we really want to teach the next generation (C1., M)

What does it mean to me? Well, it’s actually a lifestyle for me … It’s not only the sport, that keeps you healthy, but this other side of it, the camaraderie, all this is really good for the soul and the spirit of our guys, aye (C2., M)

Yeah, it’s just like bringing others onto the journey, and they will benefit too eh, sharing the load, picking each other up …it motivated me, it made me accountable, it made me accountable that I had to do my part for us to succeed … so yeah it was all about commitment, being committed, being accountable (C3., F)

Kotahitanga is a term that refers to collective unity and cohesion (Barlow, 1991). Recognizing the diversity within whānau and among individual members, kotahitanga encourages an attitude of inclusiveness and cooperation, establishing a common sense of belonging and solidarity with one another and with the environment. The case studies demonstrate the benefits of intergenerational interaction and the increased social cohesion participants experienced because of their involvement.

I like the taha Māori [oneness] that is practiced here, it’s part of marae life…you have babies, you have children, you have parents, you have grandparents, and in our lucky situation we have great grandparents, and so those are the aspects that we really look forward (C1., M)

It brings a really strong rhythm into your life because you’re in a team, so you have to turn up to practice and you’ve made that commitment, and I have this amazing group of women that I paddle with that are just, they are all sorts of ages and all sorts of people, and we just, we really get there. There’s just really beautiful gelling of who we are, and we have a lot of fun, we’re quite silly sometimes, and that’s really good when you’re getting older (C2., F)

All of this just brings us closer together and it keeps that bond and that kotahitanga…I feel safe and wanted and welcome and that we’re all in it together (C3., F)

Kaitiakitanga acknowledges that health is inextricably linked to the environment. In this framework, kaitiakitanga emphasizes reciprocity with the environment, encouraging whānau to recognize their responsibility as kaitiaki (guardians) of the natural environment This was evident across the case studies with whānau being provided with different opportunities to experience and strengthen that relationship first-hand.

It’s that engrained, cultural identity, that if we don’t have a connection to some land, somewhere, then we’re lost (C1., M)

Well, it is a lifeforce. If we stuff it up, we’re in trouble. Last time I went out on a single [waka], I came back and filled a plastic container…because it was all floating around so we just picked it up (C2., M)

I feel a stronger connection to the environment… you know I see the greenery, I hear the tui, so yeah, it is lovely, it is nice, and it makes you, for me it makes me pay more attention to nature…you become more in tune, more in tune with nature (C3., F)

### Ngā hua (the fruits): represents outcomes experienced by whānau

While ngā rākau (values) laid the foundation for whānau experiences, ngā hua (fruits of success) discusses the key outcomes experienced by whānau. Common outcomes experienced by whānau engaging in these whānau-centred initiatives can be characterized as whakamana, he taonga tuku iho and toiora. Whakamana is a concept about enabling and empowering potential (Durie, 2011). The case studies demonstrate that when whānau feel supported and valued, it can help them develop a sense of self-confidence and belief in their own abilities.

I think as well that confidence has grown because we’re surrounded by our ancestors…through the encouragement of our whānau hui I’ve noticed that we’re more confident and we’ll stand up and talk and say who we are and where we’re from and what we’re doing, that’s a big, big step for us (C1., F)

It’s a very inclusive environment…I feel like I’m fitter and stronger than I’ve been all my life really. And it’s [given] me a huge amount more confidence in myself (C2., F).

Because I became more confident and healthier, I felt better about myself…last summer I went to the beach a few times…I just used to not sort of do things like that so yeah that really stems from the whānau tri, it’s just that confidence for me has been the most amazing thing (C3., F).

He taonga tuku iho refers to ancestral treasures that have been transmitted through successive generations, providing a sense of identity and continuity (Marsden and Royal, 2003). In this context, he taonga tuku iho represents the ability for whānau to secure their cultural identity. This was evident in two of the case studies where whānau spoke about how the experiences of the whānau-centred initiative helped secure their cultural identity as Māori.

I love learning the new waiata, the new songs, and to listen to the kids doing the haka and to listen to them do their pepeha and I watch them feel very comfortable being in Parewahawaha and staying there and learning, and these are the big benefits that I see (C1., F).

I just like coming together and I feel more connected to my Māori side (C1., F).

It’s given me a doorway into the Māori world (C2., F)

You know, it’s part of what we do, we need to appraise ourselves of local customs and stories of the local iwi [tribe] and hapū [subtribe], so yeah, so that’s a more active sphere for me exploring that (C2., F)

While there was no explicit discussion of cultural identity among whānau in the third case study, whānau did recognize the benefits of participating in a programme based on te ao Māori values.

I actually just really loved the whole family whānau style of it. It’s not about who’s better or whose got the flashiest stuff. It’s just about everyone being included and everyone doing it, you know (C3., M)

It’s kotahitanga, whanaungatanga and it’s all of that encompassing into one (C3., F)

Toiora is a Māori concept encompassing holistic wellbeing and balance across physical, mental, spiritual and social aspects of life (Moorfield, n.d. b). It emphasizes the pursuit of enduring wellbeing, rooted in cultural identity, connection to land, and a vibrant sense of vitality and purpose. In this context, it is used to discuss beneficial lifestyle changes whānau have made as a result of their participation in the whānau-centred initiative.

You feel like you’re doing something to keep the revitalisation of the Māori language and culture going, I feel like that’s something that we’re doing in amongst our hui… it’s a chance to incorporate te reo Māori into everything that we do (C1., M)

I feel like I’m fitter and stronger than I’ve been all my life really…it’s an exercise without thinking you’re exercising (C2., M)

It’s a lifestyle change, and then my whole entire whānau went on this change (C3., F).

Just seeing all those unhealthy things that have been left behind, no cigarettes, no alcohol, um, just such positive role models, you know, they’re pretty amazing (C3., F)

### Ngā manu (the birds): represents the seed dispersal process, which allows for new growth and self-determination

It is through the birds who eat the fruits of the tree and disperse the seeds, that kahikatea trees are propagated (Burke, 1974; Dawson *et al.*, 2020). Likewise, the seeds of achievement sown through the participation in these whānau-centred initiatives are dispersed throughout the whānau community, providing new opportunities for growth. This aligns to the concept of mana motuhake, or Māori self-determination, which can be defined as the ability to choose one’s own path in life through increased self-reliance.

I think these hui can take us anywhere, anywhere that we want to go with it, I think it can take us there (C1., F)

It’s really challenged me in lots of ways, all the different relationships, different types of people, being on the committee, so it’s brought a lot of growth in lots of ways (C2., F)

I loved the whānau tri, I just loved it, so I loved going and then from there I was like yeah I’m actually enjoying it, I’m liking it, so I’m carrying on this journey now, you know I actually look forward to going to Crossfit (C3., F)

It [whānau tri] was a ricochet of ‘what can I do now? (C3., F)

## DISCUSSION

The success of these case studies redirects our attention from a narrow clinical approach towards a holistic perspective of health that is more in keeping with Indigenous health practices. By emphasizing the foundational values that underpin successful whānau initiatives, the Tū Kahikatea framework demonstrates the connection between values and outcomes, as well as the way in which whānau-centred initiatives can pave the way for whānau self-determination. For example, if projects are embedded in foundations that are relevant to whānau, they are more likely to experience favourable outcomes, which are more likely to help them reach a level of self-determination.

Throughout these case studies, values such as whanaungatanga, manaakitanga, kotahitanga and kaitiakitanga played an important role in engaging and retaining whānau. Numerous studies, including those on the advantages of kapa haka (Pihama *et al.*, 2014; Thompson *et al.*, 2017) and Māori participation in physical activity (Pohatu, 2015; Warbrick *et al.*, 2016) substantiate these findings. Clinical studies examining strategies to improve the lives of cancer survivors (Koia, 2019; Kidd *et al.*, 2020) as well as research on how whānau perceive encounters with neurorehabilitation (Elder, 2017; Wilson *et al.*, 2021a) also provide additional evidence for the importance of foundational values discussed in these case studies. This highlights the importance of developing authentic and respectful relationships with whānau to secure their participation, as well as the importance of developing programmes that are led by whānau and based on core values that are relatable to participants.

As a result of the core values that underpinned these initiatives, whānau gained social, cultural and health benefits, including whakamana (enhanced self-confidence), he taonga tuku iho (secure cultural identity) and toiora (enduring wellbeing). Whānau gained confidence in their abilities as a result of their efforts, which increased their self-esteem and sense of self-worth. More opportunities for healthy living and social participation opened up for whānau as they gained the self-confidence to use new services or take on leadership roles within their whānau and/or community. This supports the findings of Masters-Awatere and Graham’s (Masters-Awatere and Graham, 2019) study, which found that participation in a kaupapa Māori-centred initiative enhanced whānau sense of self-determination and confidence in accessing different health services. Additionally, the case studies illustrate practical ways in which whānau were given the opportunity to participate in te ao Māori, thereby securing their cultural identity. This builds on research showing how secure cultural identity can protect Māori against a range of negative outcomes such as depression, suicidality and economic hardship, all of which are decreased when one has a strong connection to te ao Māori (Durie, 2001; Waiti and Kingi, 2014; Houkamau and Sibley, 2015). Whānau also discussed making healthier lifestyle choices, such as limiting sugary drinks, increasing their exercise levels and incorporating te reo into their daily lives. These measures instilled in whānau a sense of self-determination and commitment to improving health outcomes showing the perpetual and sustainable nature of these types of initiatives.

From a te ao Māori perspective, self-determination can be referred to as mana motuhake which emphasizes collective determination and autonomy in defining problems and identifying solutions (Motu, 2020; Oetzel *et al.*, 2020). These case studies, like those from Te Pūtahitanga o Te Waipounamu (Savage *et al.*, 2018, 2020, 2021) and Te Whānau o Waipareira (Te Pae Herenga o Tāmaki, 2017) show whānau expressing mana motuhake by developing their own ambitions and devising ways to achieve them. Increased personal agency and control over one’s life is associated with better health and social results, as evidenced by studies demonstrating how improving whānau self-determination results in greater whānau wellbeing (Murphy, 2014; McMeeking and Pierre, 2019). As a result of colonization processes that gradually eroded Māori self-determination, whānau now need to re-establish self-determination as a cultural practice in their own respective contexts (McMeeking *et al.*, 2020). These case studies show how bottom-up, grass-roots efforts can assist whānau communities reclaim a sense of self-determination.

Mana motuhake has been identified as a fundamental element in New Zealand’s health system, with both the Māori Health Action Plan (Ministry of Health, 2020) and the newly constituted Māori Health Authority (Department of the Prime Minister and Cabinet, 2022) using the term as a crucial goal. The premise is that these reforms will enable Māori to build systems and health solutions that are beneficial to Māori. Historically, government health and social services for Māori have been developed with an emphasis on individuals and single-issue concerns, rather than on whānau as a collective (McMeeking and Pierre, 2019). Additionally, kaupapa Māori services have been hindered by contractual agreements with the government that are prescriptive, fragmented and compliance driven (Durie *et al.* 2010; McMeeking *et al.*, 2020). The current health reforms in Aotearoa New Zealand present an opportunity to reflect on past shortcomings and to commit to doing things differently going forward.

These case studies illustrate that whānau have the ability to effect positive change not only in their personal situations, but also in the development of their whānau and broader communities. This highlights the importance of funding contracts for Māori health services that align with the overarching focus on empowering whānau to take control of their own health and wellbeing, in a manner that enhances the mana motuhake of whanau.

This supports the concept that service design and delivery should be centred on the requirements of whānau and the people who use them, rather than a top-down conventional approach in which health practitioners determine what those needs look like. While not all solutions will be the same, this approach recognizes that whānau and their circumstances differ, and that whānau must be fully engaged in creating goals and capitalizing on their strengths and resources in order to achieve their objectives. As a result, service development should aim to provide whānau with the assistance and resources needed to identify and define the thriving pathways that are unique to their individual whānau and/or community.

## CONCLUSION

This study highlights the diversity and ways in which whānau-centred initiatives increase whānau capacity, emphasizing the critical importance of whānau in enhancing Māori health and wellbeing. Consistent with an Indigenous approach to health promotion, this research indicates that interventions that take into account Māori worldviews and values, as well as those generated within Māori communities, will have a greater influence on Māori health outcomes than top-down, single-issue public health promotion programmes. It adds to the global call for more expansive thinking and the integration of Indigenous viewpoints and practices into Westernized healthcare to improve Indigenous health outcomes. The Tū Kahikatea framework presented here can be used to foster the development of initiatives anchored in te ao Māori focussing on whānau strengths and self-determination rather than prevalent deficiency narratives. Rather than focussing on problems, these types of efforts will help whānau thrive. Whilst recognizing that the impact of colonization continues to have negative repercussions for whānau wellbeing, this perspective highlights enormous and collaborative capabilities that can be mobilized within whānau to address these challenges. With the evolving landscape of the health system in Aotearoa New Zealand, we have a unique opportunity to embrace and further enhance whānau ora practices that empower and uplift whānau. It is important that we continue to advocate for and prioritize practices that acknowledge and reinforce the inherent strengths, resilience and potential of whānau in order to achieve positive, sustainable change. In this way, we can foster a culture of resilience and self-determination within whānau, leading to increased mana motuhake and improved overall wellbeing.
